# A Crucial Test of Homœopathic Medicines

**Published:** 1880-07

**Authors:** 


### A CRUCIAL TEST OF HOMŒOPATHIC MEDICINES.

In the New York *Homœopathic Times* for March, 1880, is an account of a series of experiments instituted for the purpose of testing the effects of the thirtieth dilution of tincture of aconite. The project was set on foot in Milwaukee by a homœopathic society and carried out with great care. In the words of the originators, "the object of this test is to determine whether or not this preparation can produce any effect on the human organism, in health or disease." "A vial of pure sugar pellets, moistened with the thirtieth Hahnemanian dilution of aconite, and nine similar vials moistened with pure alcohol, so as to make them resemble the test pellets," were given to the prover, who was not to know which of the ten vials contained the aconite. The vials were numbered from 1 to 10, and the prover was to administer them to individuals, sick or well, and to detect by the effects which of the vials contained the medicine. It was provided that "the provers must be physicians of decided ability, who possess a good knowledge ofthe recorded symptomatology of aconite, and who have faith in the efficacy of the thirtieth dilution." The project was widely announced, and the ten-vial package was sent to each of twenty-five homœopathic physicians applying for them, scattered over a dozen different States. To guard against all possible fraud or trickery, the Rev. Geo. T. Ladd, Professor of Mental and Moral Philosophy in Bowdoin College, Maine, was selected to distribute the vials to applicants, and to receive reports from them.

Now, all this was not only decidedly fair, but it was highly creditable to those who ventured on an experiment involving so much peril to a favorite theory. One looks to the result with much interest. The result, so far as it has transpired, appears in the report of Mr. Ladd, which was not made until after the date allowed for the returns from the provers. By his report it appears that only nine of those gentlemen ventured on any answer whatever. Mr. Ladd's report is thus summarized in the general report made to the Milwaukee Academy of Medicine—the body which originated the project—and signed by Samuel Potter, M. D., President, and Eugene F. Storke, M. D., Secretary:

| | |
|---|---|
| Number of tests applied for and sent out, - - | 25 |
| Number of tests which have been reported on, - - | 9 |
| Number of tests in which the medicated vial was found, | 0 |

Be it remembered that these statements do not come from the opponents of homœopathy, but from its own adherents, and not from a local or partial source, but from a select body representing the more intelligent portion of the sect. We have never met with any evidence more damaging to homœopathy. True, the blow strikes only at the infinitesimal phase of the system, and not at the dogma of simila similibus; but it is also true that the head and front of homœopathy is the unphilosophical, unscientific and absurd doctrine of potentization, and not the theory implied by its title.

We have observed no notice of this report except in the journal named. It would appear that a general effort has been made to suppress it. In the meeting of the New York State Homœopathic Society, lately held at Albany, the report was refused acceptance. The editor of the *Times* complains of this, saying that common courtesy required its reception, though its adoption might have been refused. We do not wonder, however, at this course. The pill was altogether too bitter for homœopathic stomachs.—*Pac. Med. and Surg. Journal.*

